# Protocol for modeling acquired resistance to targeted therapeutics in adherent and suspension cancer cell lines via *in situ* resistance assay

**DOI:** 10.1016/j.xpro.2024.103361

**Published:** 2024-10-05

**Authors:** Nancy E. Sealover, Jacob M. Hughes, Patricia L. Theard, Deepan Chatterjee, Amanda J. Linke, Bridget A. Finniff, Brianna R. Daley, Robert E. Lewis, Robert L. Kortum

**Affiliations:** 1 Department of Pharmacology and Molecular Therapeutics, Uniformed Services University of the Health Sciences, Bethesda, MD 20814, USA; 2 Eppley Institute, Fred & Pamela Buffett Cancer Center, University of Nebraska Medical Center, Omaha, NE 68198, USA

**Keywords:** Cell Biology, Cell culture, Cell-based Assays, Cancer, High-Throughput Screening

## Abstract

Acquired resistance to oncogene-targeted therapies is the major driver of mortality for patients with cancer. Here, we present a 6-to-16-week assay to model the development of acquired resistance in adherent and suspension cancer cell lines. We describe steps for determining therapeutic dose, assaying acquired resistance, and testing combination therapies. This protocol is a high-throughput, cost-effective, and scalable method to model acquired drug resistance to established and newly developed therapies.

For complete details on the use and execution of this protocol, please refer to Sealover et al.^1^ and Theard et al.^2^

## Highlights

• Acquired resistance to oncogene targeted therapies can be modeled *in situ* by ISRA
• *In situ* resistance assays are high throughput, cost effective, and scalable
• Resistant populations can be isolated from *in situ* resistance assays (ISRAs)
• ISRA can be adopted to test combinations designed to limit acquired resistance

## Abstract (editor highlights)

Publisher’s note: Undertaking any experimental protocol requires adherence to local institutional guidelines for laboratory safety and ethics.

## Graphical abstract

**Figure:**
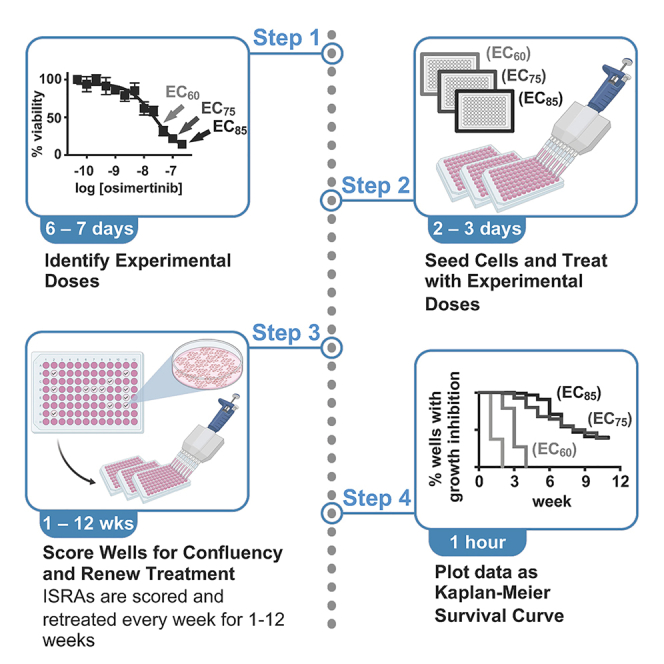

## Before you begin

Intrinsic and acquired resistance to oncogene-targeted therapies limits their overall effectiveness for treating patients with cancer. Most studies assessing combination therapies focus on enhancing therapeutic effectiveness to overcome intrinsic resistance, however, methods to assess acquired resistance are lacking. Here, we combined elements of extended proliferation outgrowth^3^^,^^4^ and time-to-progression^5^ assays to develop an *In Situ* Resistance Assay (ISRA). ISRAs model the development of acquired resistance over a 6- to 16-week time frame. The protocol presented here describes how to model acquired resistance to the 3^rd^-generation EGFR-TKI osimertinib in *EGFR*-mutated lung adenocarcinoma cells. Using this assay, we have successfully modeled acquired resistance to a number of EGFR-RAS pathway targeted therapeutics including osimertinib, the KRAS^G12C^ inhibitors adagrasib and sotorasib, the MEK inhibitor trametinib, and the farnesyl-transferase inhibitor (FTI) tipifarnib in multiple cancer cell lines.^1^ This protocol first provides step by step instructions for conducting ISRAs in 2D adherent cell culture.

We further present two extensions to the 2D ISRA described in Sealover et al.^1^ First, we describe modification of the 2D ISRA that both allows modeling of acquired resistance in 3D cultures of non-adherent cell cultures and assesses acquired resistance to conventional chemotherapeutic agents. Second, we describe how to use the ISRAs to assess secondary therapeutic targets whose inhibition might limit the development of acquired resistance to oncogene-targeted therapies. Using this model system, we showed that either SHP2^1^ inhibition or *SOS2*^*KO,*^^2^ delayed the onset and reduced the frequency of populations that could become osimertinib resistant. The ISRA is an affordable, scalable method to model acquired therapeutic resistance and assess strategies designed to overcome this resistance in cancer cell lines.

### Identify experimental doses that will be used in ISRAs

**Timing: 5 days, 1–4 h per day**

Here, you will determine the EC_50_, EC_60_, EC_75_, EC_80_, and EC_85_ values of your compound(s) in each cell line of interest before proceeding to ISRA experiments. If these values have already been determined in the cell line you will be using, you may skip this preliminary step and move to the main protocol.

**Table 1:** Recommended doses for compound dose curves

	Tube 1	Tube 2	Tube 3	Tube 4	Tube 5	Tube 6	Tube 7	Tube 8	Tube 9	Tube 10
Targeted agents	Control, no tx	0.0001 nM	0.001 nM	0.01 nM	0.1 nM	1 nM	10 nM	100 nM	1,000 nM	10,000 nM
Conventional chemotherapeutics	Control, no tx	3 nM	10 nM	30 nM	100 nM	300 nM	1,000 nM	3,000 nM	10,000 nM	30,000 nM

#### Day 1

**Timing: 1–2 h**

1. Prepare white-wall 96-well tissue culture plates by adding 200 μL of PBS to the edge wells (10 min).

***Note:*** Only the inner 60-wells (6 rows × 10 columns) are used in dose response curves to avoid edge effects due to evaporation. PBS in the outer wells ‘buffers’ edged effects.

2. Prepare cell lines for plating.
  a. Wash cells with sterile 1× PBS.
  b. Add enough trypsin-EDTA (typically 1 mL in 10 cm dish) to completely cover the cells.
  c. Place at 37°C for 5–10 min.
  d. Check under the microscope that cells have detached from the plate.
  e. Add 9 mL of complete media slowly to stop the trypsin-EDTA reaction. Pipette up and down several times to achieve a single cell suspension and avoid clumping of cells.
  f. Collect all cells in a sterile 50 mL conical tube.
  g. Count cells using a hemocytometer by trypan blue exclusion.

**CRITICAL:** Only cultures showing >85%–90% viability at time of seeding should be used. If viability is less, adjust culture conditions to ensure you have viable cells.

3. Plate cells for dose response curves.
  a. Dilute cells to 2500 / mL for adherent cell lines or 10,000 / mL for suspension cell lines in complete media.
  b. Seed 100 μL of cells per well in the inner 60-wells of the 96-well plate.

***Note:*** Further optimization of cell densities may be required depending on parameters such as cell duplication time.

4. Allow cells to incubate overnight (16–24 h) at the appropriate incubator conditions for each cell line (most commonly set at 37°C and 5% CO_2_).

#### Day 2

**Timing: 2–4 h**

5. Prepare compound dose curve in media appropriate for the cell line at a 2× concentration. Add the volume of solvent (usually DMSO) equivalent to volume added to the highest compound dose (Tube 10) to the control tube (Tube 1) (30–60 min).

**CRITICAL:** The recommended compound doses listed above are *final* concentrations in each well. Drugs are prepared at a 2× concentration and then added on top of the previously plated cells as a 1:1 dilution. As an example, 10 mM osimertinib (in DMSO) is diluted 1:500 in RPMI (complete medium) to obtain a 2× concentration for tube #10. Subsequent tubes are serially diluted from Tube #10.

**CRITICAL:** When preparing serial dilutions of compounds, make sure to change the pipette tip for each subsequent dilution. Failure to do so may result in carrying excess drug into lower dose vials.

***Note:*** We recommend plating the most concentrated dose of the compound farthest away from the control within the 96-well plate to avoid unintentional effects on the control well.

***Note:*** These are recommended doses based on our own experience with the compounds used and doses may need to be varied by cell line model and compound.

***Note:*** Drugs can be made up the day before and stored at 4°C. Drugs are then warmed to at 37°C before use.

***Note:*** As an alternative to only controlling for solvent in the control wells, one can prepare the entire dose curve at 1000× in solvent (usually DMSO), and then dilute each concentrated dose 1:500 in complete media prior to drugging cells.

6. Plate 100 μL of each 2× concentrated dilution as a dose-response curve on the cells in your 96-well plates on top of the 100 μL containing cells (200 μL final volume in each inner well, note there is no media change).

***Note:*** Using a multichannel pipette and a 12-well reservoir (one well for each compound dilution) optimizes this process.

***Note:*** We recommend plating in the orientation(s) shown in Table 1, with each dilution plated in triplicate rows. This allows for 2 dose curves to fit on one 96-well plate.

7. Allow the cells to incubate with the compound for 72 h at the appropriate cell culture conditions for each cell line (most commonly set at 37°C and 5% CO_2_).

***Note:*** The most common incubation time before collecting cell viability data for dose response curves in our experience is 72–96 h, however this may vary by compound and by field of research.

#### Day 4

**Timing: 5 min**

8. Place CellTiter-Glo 2.0 aliquot wrapped in foil (to protect from light) on bench overnight (8–16 h) to allow it to equilibrate to at 20°C–25°C (RT).

**CRITICAL:** CellTiter-Glo 2.0 should never be heated at 37°C. Bring to 20°C–25°C by either leaving an aliquot out overnight or by placing in 20°C–25°C water bath for 2 h.

#### Day 5

**Timing: 2–4 h**

9. Collect cell viability read outs for each well.
  a. Remove plate(s) from incubator and place on bench for 30 min to allow to come to 20°C–25°C (RT).
  b. Add 25 μL of CellTiter-Glo 2.0 to each well and incubate for approximately 45–60 min without exposure to light at 20°C–25°C. This is done in a drawer at bench.
    ***Note:*** The CellTiter-Glo 2.0 manual recommends a 1:1 ratio of reagent to media, however we have determined that further dilution of the reagent still produces reliable data through optimization experiments. We therefore recommend testing if increased CellTiter-Glo 2.0 dilution gives linear results at several cell concentrations (250–20,000 cells) in each cell line prior to dilution past 1:1.
    ***Note:*** Other methods of assessing cell viability (MTT assay, alamarBlue) may be used. In our experience CellTiter-Glo 2.0 gives the most robust, repeatable results.
  c. Read the luminescence of each well using a plate reader.
    ***Note:*** We use an Agilent BioTek Cytation 5 Cell Imaging Multimode Plate Reader with the following settings: emission (full light), optics (top), gain (135), read speed (normal), delay (100 ms), extended dynamic range, read height (4.5 mm).
  d. Export spreadsheet and normalize treated well values to control untreated well values.
10. Use GraphPad Prism software to calculate EC_50_, EC_60_, EC_75_, EC_80_, and EC_85_ values.
  a. Set the average of untreated cells to 100, calculate the percent survival for each well based on the formula (experimental well / average (untreated wells)).
  b. Paste Data into an XY plot in GraphPad Prism with Y = 3 replicates in side-by-side subcolumns.
  c. Find EC_anything_ values by clicking analysis > XY analysis > nonlinear regression (curve fit) > Dose-response-Special > X is log(concentration) – log(agonist vs. response—Find ECanything.

**CRITICAL:** You must click on ‘constrain’ tab and set F constant or equal to 100 – the EC_X_ value you want. For example, to find EC_80_, set F to 20.

**Figure 1:**
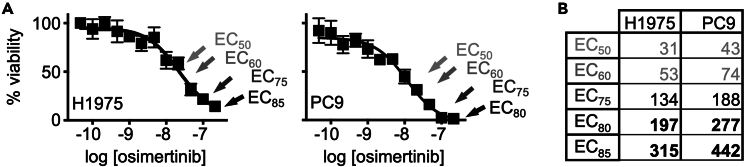
Determination of the appropriate doses of osimertinib to use in ISRA (A and B) Dose response curves (A) and EC_50_ – EC_85_ values (B) for *EGFR*-mutated H1975 (right) and PC9 (middle) cells treated with increasing doses of osimertinib on a semi-log scale from 10^−10.5^–10^−6.5^ for 72 h. Data are from Sealover et al. (2024).^1^ Data are represented as mean +/− SD from three independent experiments.

In the example presented in Figure 1, the EC_50_ - EC_85_ for osimertinib in H1975 and PC9 cells was 30–450 nM.

## Key resources table

**Table:** 

REAGENT or RESOURCE	SOURCE	IDENTIFIER
**Chemicals, peptides, and recombinant proteins**		

Osimertinib (AZD9291; 3^rd^ gen EGFR inhibitor) 10 mM (1 mL in DMSO)	Selleck Chemicals	Cat# S7297; CAS: 1421373-65-0
Cisplatin (DNA synthesis inhibitor)	Selleck Chemicals or similar (e.g., MedChemExpress, Sigma)	Cat# S1166; CAS: 15663-27-1

**Critical commercial assays**		

CellTiter-Glo 2.0	Promega	Cat# G9243

**Experimental models: Cell lines**		

NCI-H1975, adherent cell line model	Obtained from Udayan Guha, available at ATCC	RRID:CVCL_UE30
HCC33, suspension cell line model	Obtained from John Minna, available at ATCC	RRID:CVCL_2052

**Software and algorithms**		

GraphPad Prism version 9.2.0	www.graphpad.com	RRID:SCR_002798
Microsoft Excel	www.microsoft.com	RRID:SCR_016137

**Other**		

96-well white-walled tissue culture plates	Revvity or similar (Thermo Fisher Scientific, Sigma, other)	Cat# 6004480
96-well clear-walled tissue culture plates	CELLTREAT or similar (Thermo Fisher Scientific, Sigma, other)	Cat# 229195
96-well clear U-bottomed ultra-low attachment plates	faCellitate BIOFLOAT or similar (Corning Costar, Thermo Fisher Scientific Nunclon Sphera, other)	Cat# F202003
NuSieve GTG agarose	Lonza	Cat# 50081
RPMI 1640 media with Glutamine XL	Quality Biological or similar (Thermo Fisher Scientific, Sigma, other)	Cat# 120-301-101
Fetal bovine serum – premium select	Bio-Techne or similar (Thermo Fisher Scientific, Sigma, other)	Cat# S11550
IMDM powder	Thermo Scientific	Cat# 12200036
Sodium bicarbonate	Sigma-Aldrich	Cat# S5761
Finnpipette Novus electronic multichannel pipette (300 μL, 12-channel)	Thermo Scientific	Cat# 1223F76
Finnpipette Novus electronic multichannel pipette (100–1,200 μL, 8-channel)	Thermo Scientific	Cat# 21-377-845
Finntip Flex 1200 pipette tips	Thermo Scientific	Cat# 94062817
Finntip Flex 300 pipette tips	Thermo Scientific	Cat# 94060513
Trypan blue solution, 0.4%	Thermo Scientific	Cat# 15250061
Penicillin/streptomycin mixture, 100×	Quality Biological	Cat# 120-095-721
50 mL reagent reservoir, polystyrene, sterile	CELLTREAT	Cat# 229291
12-channel reagent reservoir, individually wrapped, sterile	Sigma-Aldrich or other (CELLTREAT, etc.)	Cat# Z370843-8EA
500 mL bottle-top vacuum filter system , 0.22 μm	Corning or other (Sigma, etc.)	Cat# 431097
BioTek Cytation 5 cell imaging multimode plate reader	Agilent	N/A

## Materials and equipment

### Reagents

#### Small molecule inhibitors

In our experience, most small molecule compounds are soluble at a 10 mM concentration in DMSO. We store our inhibitors as 50 μL aliquots at −20°C. This allows us to prevent multiple freeze-thaw cycles. Solubility and storage may be different for certain small molecules, we recommend always consulting the manufacture’s website or product information for solubility and storage conditions.

#### Reagents for ISRAs for non-adherent (suspension) cell lines

**Table:** Preparation of 1.6% soft agar for suspension cell model (3D) ISRAs

Reagent	Final concentration	Amount
NuSieve GTG Agarose	1.6%	3.2 g
Sterile H_2_O	–	200 mL

Autoclave agar and sterile water together after mixing.

***Note:*** 1.6% soft agar mixture should be placed in a 40°C water or bead bath 3–4 h before plating cells to ensure it is in a liquid state.

**Table:** Preparation of 2× IMDM complete media for suspension cell model (3D) ISRAs

Reagent	Final concentration	Amount
IMDM powder - packet	2×	1 packet
Sodium bicarbonate	72 mM	3.024 g
Sterile H_2_O	–	Up to 500 mL

Start by dissolving IMDM powder and sodium bicarbonate in 400 mL water and mix with stir bar until completely dissolved. Rinse IMDM powder packet with water to ensure you get all of the contents. Bring to total volume of 500 mL and filter sterilize.

## Step-by-step method details

### Seed cells and treat with EC_50_, EC_60_, EC_75_, and EC_80_-EC_85_ compound concentrations to begin ISRA experiments

**Timing: 2–3 days**

This section describes how to begin your ISRA experiments using the EC values determined in ‘before you begin’. The protocol is written primarily for use in 2D (adherent) cultured cells. When steps are different between 2D versus 3D cultures, they are enumerated separately.

***Note:*** We recommend only performing 3D ISRAs on cell culture lines that are non-adherent (suspension cultures). This recommendation is to avoid selecting for ‘aggressive’ clones.

**Figure 2:**
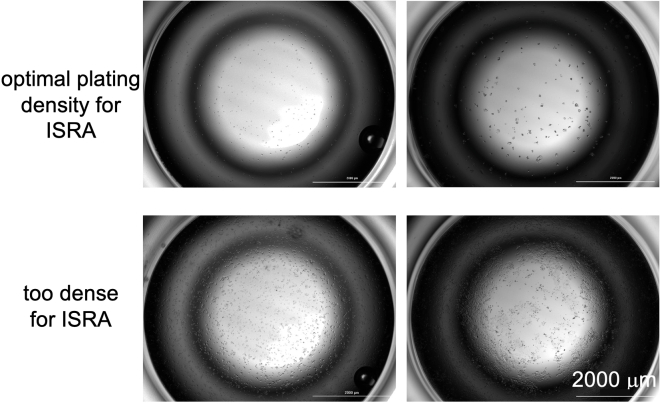
Examples of appropriate seeding density for ISRA, related to step 2 1.25× images of wells seeded with increasing densities of H1975 cells in a 96-well plate and allowed to rest for 24 h. (Top) cells seeded at 150 or 250 cells / well, showing an optimal density for beginning drug treatment for an ISRA. (Bottom) cells seeded at 500 or 750 cells / well, showing >15% confluency and thus being too dense to start treatment for an ISRA.

**Figure 3:**
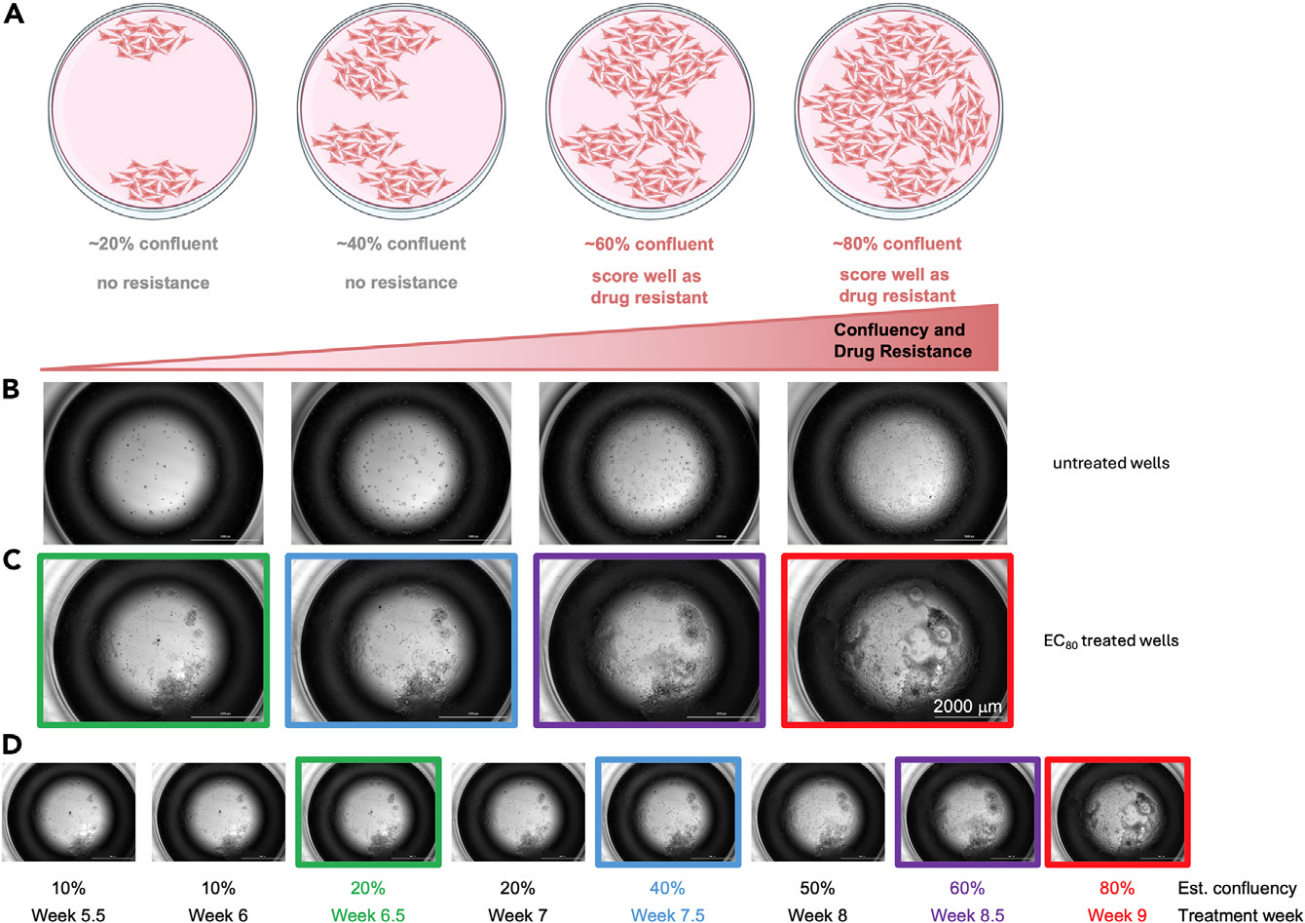
Assessment of confluence during ISRA, related to step 5 (A) Schematic representation of well at 20%–80% confluence. (B) Images of cells plated for an ISRA and left untreated for 4–14 days showing cells at 20–80% confluence. (C and D) Serial images of a single well treated with an EC_80_ dose of oncogene- targeted therapy. (C) shows examples of the well at 20% (green box), 40% (blue box), 60% (purple box), and 80% (red box) confluency. (D) shows the same well imaged every 3–4 days from week 5.5 (day 39) to week 9 (day 72) of the ISRA. Colored boxes correspond to the identical images in C and D.

**Figure 4:**
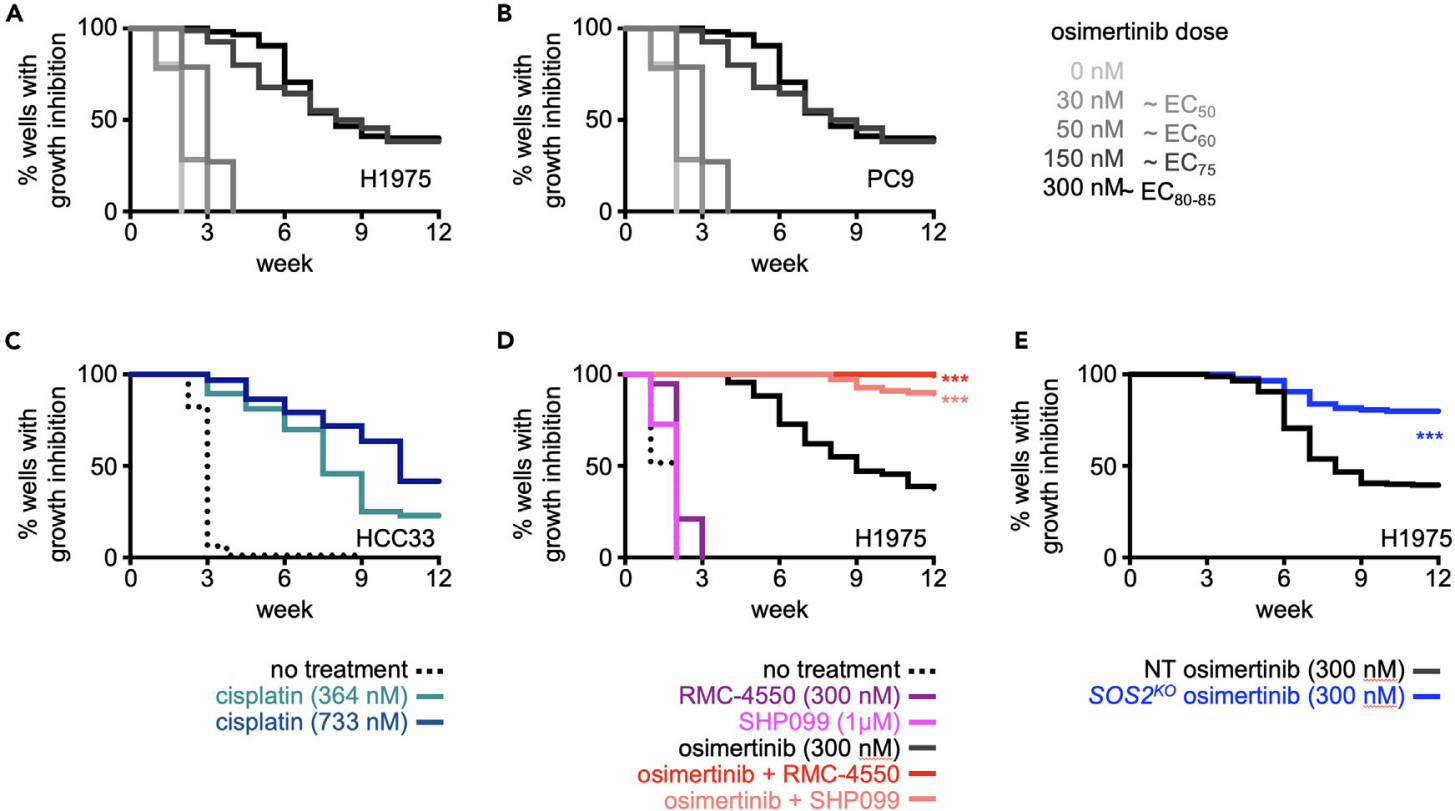
Assessment of acquired osimertinib resistance by ISRA (A and B) Kaplan-Meier survival curves showing ISRA assay data for *EGFR*-mutated H1975 (A) or PC9 (B) cells treated with the indicated osimertinib doses equivalent to EC_50_, EC_60_, EC_75_, and EC_80-85_ doses of osimertinib as assessed in Figure 1. Data are from Sealover et al. (2024).^1^ (C) Kaplan-Meier survival curves showing ISRA assay data for HCC33 cells treated with the indicated cisplatin doses under 3D culture conditions. (D) Kaplan-Meier survival curves from ISRAs in which H1975 cells were left untreated (black, dashed) or treated with 300 nM osimertinib alone (black), a SHP2 inhibitor alone (RMC-4550 at 300 nM or SHP099 at 1 μM) (purples), or the combination of osimertinib + RMC-4550 or SHP099 (reds). These data show the extent to which an EC_50_ dose of two distinct SHP2 inhibitors limit the development of osimertinib resistance and are from Sealover et al. (2024).^1^ (E) Kaplan-Meier survival curves from ISRAs in which NT (black) or *SOS2*^*KO*^ (blue) H1975 cells were treated with 300 nM osimertinib. These data show the extent to which *SOS2*^*KO*^ limits the development of osimertinib resistance and are from Theard et al. (2024). SOS2 modulates the threshold of EGFR signaling to regulate osimertinib efficacy and resistance in lung adenocarcinoma / Molecular Oncology 18 (3) was published under a Creative Commons Attribution (CC BY) License. A link to this manuscript can be found at: https://febs.onlinelibrary.wiley.com/doi/full/10.1002/1878-0261.13564. Data for Kaplan-Meier survival analysis are pooled from three independent trials. ∗∗∗*p* < 0.001 compared to osimertinib-treated cells.

Methods video S1. Feeding ISRA assays, related to step 7 The video shows the proper techniques for preparing the area for feeding ISRA assays, removing media from ISRA plates by inversion into a prepared containment tray, and feeding of ISRA plates.

#### Day 1

**Timing: 1–4 h**

1. Prepare cell lines for plating.
  a. Determine the number of 96-well plates you will need for your experiment(s).
    ***Note:*** Your first experiment for a single compound in a single cell line will include a control plate and four treatment plates (five plates total).
  b. Collect cells from a 10 cm tissue culture dish (adherent) or T-75 flask (suspension) into a 50 mL sterile conical tube, pipetting to achieve a single cell suspension as outlined above.
  c. Count your cells using an automated cell counter or hemocytometer by trypan blue exclusion. Only use cultures showing >85%–90% viability at time of seeding.
  d. Dilute cells to 2500 / mL for adherent cell lines or 20,000 / mL for suspension cell lines in complete media.
    ***Note:*** You will need to prepare 12 mL / plate for adherent cells and 6.5 mL / plate for suspension cells. The excess volume is to account for volume losses during plating. For example, prepare 60 mL of cells for a 5-plate experiment with adherent cells.
    **CRITICAL:** Move immediately to the next step. It is essential to plate cells in a timely manner to ensure that each well of the 96-well plate receives the correct number of cells. We recommend inverting the tube containing the diluted cells often, to ensure the cell dilution mixture remains homogenous.
2. Seed cells in a 96-well clear walled tissue culture plate.
  a. **Adherent cells**: Plate 100 μL of cells in each well of the plate using a 100–1200 μL electronic multichannel pipette or manual multichannel pipette and a 50 mL reservoir.
  b. **Suspension cells:** Dilute cells to 10,000 / mL in 0.4% agar; plate 100 μL of cells in each well.
    i. Dilute the 1.6% soft agar reagent with 2× complete IMDM media in a 1:1 volumetric ratio to make 0.8% soft agar. Keep at 40°C.
    ii. Mix the 0.8% soft agar dilution with the diluted cells from STEP 1 in a 1:1 volumetric ratio. Plate 100 μL of the cell and soft agar mixture into each well immediately to achieve a final concentration of 1000 cells/well.
  c. Allow cells to incubate for 24–48 h.
    i. **Adherent cells:** Place plates in the incubator for 24–48 h to allow cells to adhere to the tissue culture plate.
    ii. **Suspension cells:** Leave plates at room temperature in the sterile laminar hood for 2–4 h to allow the mixture of agar and cells to solidify. Then place cells in incubator for feeding the next day.

***Note:*** We typically drug cells 24 h after seeding, however, we have encountered a few cell lines that are slow to adhere. In this case, we wait 48 h before adding drug.

**CRITICAL:** Cells should not be more than 10%–15% confluent after seeding. If your cells are more than 15% confluent after seeding, you need to replate your cells or try a lower density of cells for that cell line model. Examples of cells plated at optimal density versus cells that are at too high of a density are shown in Figure 2.

#### Day 2 or day 3

**Timing: 1–4 h**

3. Treat cells with EC_50_, EC_60_, EC_75_, and EC_80_-EC_85_ compound concentrations.
  a. Prepare a 10 μM working stock of each compound by diluting the 10 mM stock 1:1000 in complete medium (1 μL of the 10 mM stock/ 1 mL of cell culture media).
  b. Label a sterile 15 mL tube for each dose of drug being prepared.
  c. Prepare 2× concentrated doses of each drug corresponding to the EC_50_, EC_60_, EC_75_, and EC_80_-EC_85_ of the compound of interest as identified in ‘before you begin’.
    i. Place the appropriate volume of media (from table below) needed to make a total of 12 mL of drug per plate.
    ii. Add the appropriate volume of 10 μM working stock to each tube.
    iii. Invert 4–6 times to mix.
      ***Note:*** For the highest dose, an ∼ EC_80_-EC_85_ can be used, depending on the shape of the dose-response curve from ‘before you begin’. For osimertinib studies, we used 300 nM which corresponded to an EC_85_ dose in H1975 cells and an EC_80_ dose in PC9 cells (Figure 1 and Sealover et al.^1^).
      ***Note:*** The table below gives both a broad reference range of potential 2× doses as well as showing preparation of 30, 50, 150, and 300 nM osimertinib for use in H1975 cells.

**Table:** 

Final drug concentration:	Volume of 10 μM stock to add to 12 mL (volume for treating one 96-well plate)	Volume of media to bring to 12 mL (approximate)
No treatment	0 μL	12 mL
1 nM	2.4 μL	12 mL
3 nM	7.2 μL	12 mL
10 nM	24 μL	12 mL
**30 nM**	72 μL	11.9 mL
**50 nM**	120 μL	11.9 mL
100 nM	240 μL	11.76 mL
**150 nM**	360 μL	11.64 mL
**300 nM**	720 μL	11.3 mL
1000 nM	2.4 mL	9.6 mL
  d. Label 96-well plates containing cells with each compound concentration. An example is provided below:

**Table:** 

Plate 1:	No treatment
Plate 2:	EC_50_ concentration
Plate 3:	EC_60_ concentration
Plate 4:	EC_75_ concentration
Plate 5:	EC_80_-EC_85_ concentration
  e. Plate 100 μL of the prepared 2× compound dilution in each well of the plate labeled with the correct corresponding concentration using a 100–1200 μL electronic multichannel pipette.
  f. Allow cells to incubate with the compound for 4–7 days before beginning to observe wells for confluency.
    ***Note:*** “No treatment” plate(s) should be monitored closely for the first 14 days to ensure cells are growing correctly independent of compound treatment. Untreated wells typically become more than 50% confluent within 14 days of the beginning of the experiment.
    ***Note:*** As outlined below, media is changed weekly in ISRA plates. There is no media change prior to scoring 1 week after plating.

### Score wells for confluency to collect data

#### Twice weekly starting on day 5

**Timing: 1–5 h, depending on the size of the experiment**

4. Prepare area for scoring.
  a. Disinfect microscope and surrounding area.
  b. Retrieve ISRA plates from incubator and place in disinfected area near microscope.
5. Score ISRAs using a light microscope with a 4× objective.
  a. Place ISRA on microscope stage starting at well B2.
  b. Adjust settings on microscope so that cells are in-focus and clear.
  c. Visually assess to determine if each well is > 50% confluent; if yes, mark on the lid above the currently viewed well to record the well as “scored”.
  d. Move to the next available, unscored row once all wells of the current row have been evaluated and repeat until all inner 60 wells have been viewed and/or scored.
  e. Record the total number of wells scored during the session, alongside the date, somewhere on the lid that will not interfere with future assessment of non-scored wells.
  f. Record the total number of wells scored per plate with the corresponding date in an Excel spreadsheet following each scoring session.
    ***Note:*** See Table S1 for a fillable excel template (tab 1) and example data assessing acquired osimertinib resistance at different osimertinib doses in H1975 cells (tab 2).
  g. Repeat 1–2 times per week until all wells are scored as resistant to treatment or the end point of the experiment is reached (12 weeks).
    ***Note:*** See Figure 3 for examples of wells either untreated or treated with an EC_85_ dose of osimertinib at different confluencies. The example in Figures 3C and 3D shows serial images of a well treated with an EC_85_ compound dose that started proliferating during week 5 the well was scored as > 50% confluent at week 8.

### ISRA treatment renewal

#### Weekly starting on day 8

**Timing: 1–4 h, depending on the size of the experiment**

6. Prepare a plastic or metal, autoclavable collection container for media being discarded from ISRAs.
  a. Place 1–4 folded paper towels in the bottom of the container.
  b. Spray the paper towels and container with 70% ethanol to completely disinfect the items.
  c. Place the disinfected collection container in the sterile laminar hood.
7. Prepare 1× EC_50_, EC_60_, EC_75_, and EC_80_ compound concentrations for treatment renewal.
  a. Prepare a 10 μM working stock of each compound by diluting the 10 mM stock 1:1000 in complete medium (1 μL 10 mM stock/mL of cell culture media).
  b. Label a sterile 50 mL (**adherent cells**) or 15 mL (**suspension cells**) tubes for each dose of drug being prepared.
  c. Pipette 24 mL (**adherent cells**) or 12 mL (**suspension cells**) of media into each tube.
  d. Remove and discard an amount of media equivalent to the amount of 10 μM working stock that will be added to each tube.
  e. Replace the discarded media with the appropriate volume of 10 μM working stock to each tube.
  f. Invert 4–6 times to mix.

**Table:** 

Final desired concentration of each well:	Volume of 10 μM stock to add to 12 mL (volume for retreating one suspension cell line model 96-well plate)	Volume of 10 μM stock to add to 24 mL (volume for retreating one adherent cell line model 96-well plate)
0 nM	0 μL	0 μL
1 nM	1.2 μL	2.4 μL
3 nM	3.6 μL	7.2 μL
10 nM	12 μL	24 μL
30 nM	36 μL	72 μL
50 nM	60 μL	120 μL
100 nM	120 μL	240 μL
150 nM	180 μL	360 μL
300 nM	360 μL	720 μL
1000 nM	1.2 mL	2.4 mL

    ***Note:*** This table is being provided as an example for reference, however your own experiments may require further calculations. For osimertinib treatments shown in Figures 4A and 4B, 30 nM, 50 nM, 150 nM, and 300 nM solutions were prepared in 24 mL.
8. Remove existing media from ISRAs and replace with newly prepared 1× treatment dilutions:
  a. Before working with adherent or suspension cell model ISRAs, prepare 50 mL reservoirs and a 100–1200 μL electronic multichannel pipette in the laminar hood.
  b. Replace existing media with newly prepared treatments:
    i. Handling plates one at a time, remove the lid of the 96-well plate corresponding to the drug treatment previously prepared in the reservoir.
    ii. In a swift, fluid motion, invert the plate over the collection container so that the existing media is removed from the wells.
      ***Note:*** If needed, complete a second downward motion to remove any large amounts of media remaining in the wells of the plate.
      **CRITICAL:** See Methods video S1: **ISRA Treatment Renewal** for a step-by-step guide to treatment renewal for ISRAs.
      ***Note:*** For suspension cells, gently invert the plate over the collection container or gently aspirate the top layer of existing media, ensuring the bottom layer of agar and cells is left undisturbed.
    iii. Quickly replace media in wells by adding the newly prepared treatment from the reservoir to the plate using the electronic multichannel pipette and return the lid to the plate.
  c. Place ISRA plates back into the incubator until the next scoring or feeding session.
  d. Aspirate media from collection container throughout the process as needed.
  e. Renew media in ISRA plates containing unscored (<50% confluent) wells once a week for the entirety of the assay.
9. Plot data as a Kaplan-Meyer survival curve using Prism.

***Note:*** An example an ISRA assessing acquired osimertinib resistance in adherent cultured H1975 and PC9 cells treated with EC_50_ - EC_85_ doses of osimertinib is given in Figures 4A and 4B.

***Note:*** An example of an ISRA in 3D cultured HCC33 (suspension cultured cells) small cell lung cancer (SCLC) cells treated with cisplatin is shown in Figure 4C.

### Additional procedure: Assessment of secondary targets to limit acquired resistance to targeted therapies by ISRA

Acquired resistance to targeted therapies limits the overall clinical effectiveness of these compounds. A critical extension of the ISRA is the ability to rapidly assess the extent to which inhibition of multiple secondary targets can delay or inhibit resistance to a give oncogene targeted therapy. These studies may be key steps in in defining new combination therapies that can prolong the therapeutic window for patients.

Assessment of secondary targets can either be performed using secondary therapeutics targeting the protein of interest or, if no compound exists for a protein target of interest, by deletion of the secondary target using CRISPR-Cas9. Secondary target selection is done by the individual laboratory based either (i) molecular profiling of resistant cultures or (ii) on their understanding of the biological system. As an example, in *EGFR*-mutated LUAD cells, acquired osimertinib resistance is driven by RTK/RAS pathway reactivation^6^ either via secondary *EGFR* mutations or enhanced signaling (via mutation or overexpression) through multiple parallel RTKs including MET, AXL, FGFR, HER2/3, and IGF-1R.^6^^,^^7^^,^^8^^,^^9^^,^^10^^,^^11^^,^^12^^,^^13^^,^^14^ Because of this, we examined proximal RTK signaling intermediates SHP2^1^ and SOS2^2^ as secondary targets whose inhibition (SHP2, Figure 4D) or deletion (*SOS2*^*KO*^, Figure 4E) might alter acquired osimertinib resistance. Additional examples assessing the extent to which SOS1 inhibition or *KSR1*^*KO*^ limits acquired trametinib resistance are in Daley et al., 2023.^15^

#### Inhibition or deletion of secondary target(s)

**Timing: variable: 1–4 weeks**

10. Identify EC_50_-EC_80_ doses for secondary compounds to be tested as outlined in ‘before you begin’.

***Note:*** When modeling the extent to which inhibiting a secondary target alters resistance to an oncogene-targeted therapy, use an EC_50_ dose of the second inhibitor. If, instead, you are modeling resistance to a secondary inhibitor alone, we recommend an EC_75_ dose of the inhibitor.

11. If there are not any small molecule inhibitors available for a given potential secondary target, the target should be deleted using CRISPR-Cas9 technology.

***Note:*** We recommend using pooled KO cells where you observed at least an 80% reduction in protein abundance compare to non-targeting controls. This is to avoid clonal effects that can occur, cell lines grown from clones often take on an aggressive growth phenotype that can obfuscate any biological effects of the KO of interest.

#### Combination ISRAs

**Timing: 6–12 weeks**

#### Day 1 (2–4 h)

12. Seed cells in multiple 96-well clear walled tissue culture plates. Seed at 250 cells / well in 100 μL.
  a. **Drug combination studies**: Seed 12 plates if testing a single secondary target; seed an additional 6 plates per additional drug target.
    ***Note:*** For the example shown in Figure 4D, we tested the extent to which two different SHP2 inhibitors (SHP2i) could limit the development of osimertinib resistance. This experiment had 18 plates, using 3 each at the following conditions:

**Table:** 

Plates 1–3	No treatment
Plates 4–6	EC_50_ of SHP2i A: RMC-4550 (300 nM)
Plates 6–9	EC_50_ of SHP2i B: SHP099 (1000 nM)
Plates 9–12	EC_85_ of osimertinib (300 nM)
Plates 13–15	EC_50_ of SHP2i A: RMC-4550 (300 nM) + EC_85_ of osimertinib (300 nM)
Plates 16–18	EC_50_ of SHP2i B: SHP099 (1000 nM) + EC_85_ of osimertinib (300 nM)
  b. **Knockout studies:** Seed 6 plates each of knockout cells and non-targeting controls.
    ***Note:*** For the example shown in Figure 4E, we tested the extent to *SOS2*^*KO*^ could limit the development of osimertinib resistance. This experiment had 12 plates, using 3 each at the following conditions:

**Table:** 

Plates 1–3	Non-targeting cells, no treatment
Plates 4–6	*SOS2*^*KO*^ cells, no treatment
Plates 6–9	Non-targeting cells EC_85_ of osimertinib (300 nM)
Plates 9–12	*SOS2*^*KO*^ cells EC_85_ of osimertinib (300 nM)

#### Day 2 (1–4 h)

13. Treat cells with compound(s) of interest.
  a. **Drug combination studies**:
    i. Prepare 4× concentrated doses of each drug corresponding to the EC_80_-EC_85_ of the oncogene-targeted therapy and the EC_50_ of any secondary inhibitors.
    ii. Treat with 50 μL of each inhibitor; use media to bring total volume in the well to 200 μL.
      For the example shown in Figure 4D, we added the following on top of the 100 μL of cells.

**Table:** 

Plates 1–3	100 μL complete media
Plates 4–6	50 μL SHP2i A: RMC-4550 (1200 nM) + 50 μL complete media
Plates 6–9	50 μL SHP2i B: SHP099 (4000 nM) + 50 μL complete media
Plates 9–12	50 μL osimertinib (1200 nM) + 50 μL complete media
Plates 13–15	50 μL SHP2i A: RMC-4550 (1200 nM) + 50 μL osimertinib (1200 nM)
Plates 16–18	50 μL SHP2i B: SHP099 (4000 nM) + 50 μL osimertinib (1200 nM)
  b. Knockout studies.
    i. Prepare 2× concentrated drug corresponding to the EC_80_-EC_85_ of the oncogene-targeted therapy.
    ii. Treat with 100 μL of either complete media (no treatment) or the oncogene-targeted therapy.

#### Twice weekly starting on day 5

**Timing: 2–8 h, depending on the size of the experiment**

14. Score and feed combination or genetically modified cell line ISRAs as described in the main protocol.

***Note:*** When feeding drug combination studies, drugs are prepared at 2× concentration and added into the appropriate plates (100 μL). For plates receiving only one compound, 100 μL complete media is added to bring the total volume up to 200 μL.

15. Plot data as a Kaplan-Meyer survival curve using Prism.

***Note:*** Example ISRAs assessing the extent to which SHP2 inhibition or *SOS2*^*KO*^ limits the development of acquired osimertinib resistance are in Figures 4D and 4E.

***Note:*** In addition to the two drug combination studies outlined above, three- and four-compound combinations can be easily tested by expanding the experiment to include the appropriate controls and combinations.

## Expected outcomes

ISRA experiments will be completed when either (i) all of the wells become greater than 50% confluent in 2- to 12-weeks or (ii) 12 (or more) weeks have passed and the experimental endpoint has been reached. At the end of the experiment, Table S1 or a similar file containing the number of wells scored each week for each treatment will contain all of the data needed to generate Kaplan-Meier survival curves to represent the data collected. Kaplan-Meier survival curves for single compound studies will resemble either Figures 4A and 4B, where multiple doses of osimertinib were tested in NCI-1975 cells under adherent culture conditions, or Figure 4C, where multiple doses of cisplatin were tested in HCC33 cells under 3D growth conditions. Increasing doses of the compound will increasingly delay the outgrowth of cells if the effect is dose dependent.

For ISRAs testing the extent to which inhibiting secondary therapeutic targets can limit the development of acquired resistance, you can expect one of two distinct outcomes. If the secondary therapeutic target is an important mediator of acquired resistance to the oncogene-targeted therapy, you will observe a delay in the time that it takes for wells to become > 50% confluent (observed as a shift to the right in the Kaplan-Meier curve) and/or a reduction in the overall frequency of wells able to become resistant to the oncogene targeted therapy. In the data shown in Figures 4D and 4E, SHP2i or *SOS2*^*KO*^ reduced the frequency of cultures that developed osimertinib resistance. If the secondary therapeutic target is not a critical driver of acquired resistance to the oncogene-targeted therapy, the combination therapy curve will overlay the curve for the oncogene-targeted therapy. An example of this can be found in Daley et al.,^15^ where we found that SOS1 inhibition did not delay acquired MEK inhibitor resistance in LUAD cells harboring *KRAS*^*Q61X*^ mutations.

Data from ISRAs can be used for a variety of purposes. For single compound experiments, dose-response experiments can be performed to both show long-term efficacy of the compound and models the development of resistance to a that compound. Here, resistant colonies can then be cultured to study therapeutic resistance. For secondary target studies, drug combination experiments can be used to test multiple combinations prior to moving into expensive animal studies. If a small molecule inhibitor is not available for a given secondary target of interest genetic knock-out cell lines can be used in proof-of-concept studies prior to proposing a protein of interest as a target for compound development.

## Quantification and statistical analysis

After entering data in Table S1, format your data as presented in tab 3, where data is converted into a vertical format compatible with making Kaplan-Meier plots in GraphPad Prism. Enter data in prism and format as desired with colors to distinguish untreated and treated groups. Export Kaplan-Meier plots from GraphPad Prism for data presentation. Statistical significance between any pair of Kaplan-Meier curves can be determined using GraphPad Prism.

## Limitations

A limitation of ISRA assays is that scoring is performed manually of each well every week, which is subjective to the individual completing the scoring. It is recommended to (i) use the included pictures in Figure 3 to train anyone completing the scoring on how to visually assess cell density and confluency within a well and (ii) have two investigators within the lab score different trials and/or take turns scoring plates to ensure that individual wells are called resistant +/− one week of becoming 50% confluent.

***Note:*** Over the past three years that we have been performing this assay, we have observed that wells that were at the ‘threshold’ of scoring (i.e., around 50% confluent) would progress well past this threshold seven days later. Thus, each individual well (of the 180 colonies scored per condition) can be thought of as +/− 1 week. Given the large number of wells scored, any scoring discrepancies or unintentional bias will largely average out over the lifetime of the assay. With practice, manual scoring is also more efficient than scoring on a plate reader and thus enhances the throughput and robustness of the assay.

***Note:*** It would be possible to further optimize this assay to automated counting on a plate reader with appropriate imaging software.

## Troubleshooting

### Problem 1

Related to step #2 (cell seeding for ISRA).

Wells in untreated control ISRA plates are reaching > 50% confluence more than 2 weeks after being plated or cells are >50% before adding treatment for the first time.

### Potential solution

• Change the number of cells seeded at the start of the experiment.
  ○ Increase cell number if untreated controls took > 2 weeks to outgrow.
  ○ Decrease cell number if untreated controls were > 50% confluent at the time of drug treatment.
• We recommend performing a set of cell dilutions across one or more plates and observe which cell dilution(s) that:
  ○ Are 10%–20% confluent before adding treatment during the first week.
  ○ Reach > 50% confluency in the appropriate time frame of 1–2 weeks, and no more than 10–20% confluent before adding treatment during the first week.

### Problem 2

Related to step #3 (drug doses used for ISRA) and step #5 (scoring ISRA).

The EC_50_ – EC_85_ values determined are insufficient to delay growth (wells in all treatment group reach > 50% confluence within 2 weeks) or cause only a modest proliferative delay rather than modeling acquired resistance (highest dosed treatment group only delays outgrowth 1–3 weeks).

This can be due to variance in a drug’s potency (related to the dose needed to observe a 50% reduction in survival) compared to efficacy (related to the amount of total growth inhibition at high doses).

In our experience, drugs that show incomplete efficacy as a single agent are more difficult to model by ISRA compared to drugs that show a >80% efficacy.

### Potential solutions

• Decrease the number of cells seeded per well to see if this extends the timeline of outgrowth in treated, but not untreated, cells. In our experience, LUAD cells are much more sensitive to RTK/RAS pathway-targeted therapies at low density (100 cells/well).
• Increase the dose of the oncogene-targeted compound up to the limit of specificity for that compound. For many new compounds, this is in the 1–10 μM range. We do not recommend going above a 10 μM dose for most compounds
• Test a second, more potent inhibitor targeting the same oncogene if one is available. For example, in Daley et al. we were unable to model sotorasib resistance at a 10 μM dose in *KRAS*^*G12C*^ mutated H2030 cells. In contrast, we were able to model adagrasib resistance at 3 μM, allowing us to assess G12Ci resistance in H2030 cells.

### Problem 3

Related to before you begin (determining EC_anything_) and step #3 (drug doses used for ISRA).

The EC_50_ – EC_85_ values determined for adherent cells delay the outgrowth of cells indefinitely, with few wells for every treatment reaching > 50% confluence within 12 weeks.

### Potential solution

• EC values obtained for 2D adherent cell lines may be inappropriate for ISRAs if the cell lines used are more sensitive to the compound at the low cell density used for this assay over an extended treatment time (>72 h).
  ○ In these instances, EC values obtained by plating dose response curves in 3D spheroid culture using ultra low attachment 96-well round bottom plates and media (agarose not necessary) over 72 h may be more accurate.
  ○ We recommend repeating dose-response curves under both 2D adherent and 3D spheroid conditions to get a range of EC_anything_ values that can be assessed by ISRA.
  ○ In our experience, LUAD cells are more sensitive to RTK/RAS targeted therapies including osimertinib,^16^ KRAS^G12C^ inhibitors,^1^ MEK inhibitors,^15^ and SOS1 inhibitors^15^^,^^16^ as 3D spheroid vs. 2D adherent cultures. This increased drug sensitivity (survival disadvantage) may more accurately reflect sensitivities seen during an ISRA. This is in contrast to survival advantages that may occur during long-term spheroid culture where aggressive clones can be selected for.

***Note:*** In Sealover et al. 2024,^1^ this trouble shooting step was necessary to obtain EC_80_-EC_85_ doses the KRAS^G12C^ inhibitors sotorasib and adagrasib to model acquired resistance in *KRAS*^*G12C*^- mutated cell lines.

• Alternatively, you can determine the appropriate dose modeling acquired resistance to a given oncogene-targeted therapy via ISRAs empirically. To do this, plate 9 × 96-well plates. Leave one plate untreated, and treat the remaining plates with increasing doses of inhibitor from 1 nM–3,000 nM on a semi-log scale (1, 3, 10, 30, 100, 300, 1000, 3000). Using this method, you will observe a small dose-dependent shift in outgrowth (1–2-week differences) at doses insufficient to model acquired resistance, and then a significant shift in the Kaplan-Meier curve with delayed outgrowth at doses sufficient to model acquired resistance.

### Problem 4

Related to step #10 (determining dose of secondary compounds to be tested in combination with an oncogene-targeted therapy).

Inhibitors of secondary targets may yield insufficient growth inhibition on their own to inhibit survival in > 50% of cells (decreased drug efficacy), thus making the determination making it difficult to determine the dose to use in combination studies.

### Potential solution

We recommend either.

• Using an EC_50_-EC_70_ dose of the drug from single inhibitor dose response curves or,
• Empirically determining the dose of the inhibitor that gives full target inhibition in a cell-based assay.

As an example, while an EC_50_ dose of the SHP2i SHP099 or RMC-4550 is insufficient to reduce survival > 50% as a single agent in H1975 cells, these same doses showed effective target inhibition (inhibition of SHP2-dependent ERK signaling).^1^^,^^17^^,^^18^^,^^19^ Because of this, EC_50_ doses of these compounds were used to examine the extent to which SHP2i could limit the development of acquired osimertinib resistance (Sealover et al.^1^; Figure 4D).

### Problem 5

Related to step #11 (deletion of secondary target using CRISPR-Cas9).

Acquired resistance to a targeted therapy in combination with deletion of a secondary target may be skewed by either (i) clonal effects independent of deletion of the gene of interest or (ii) incomplete KO of the secondary target.

### Potential solution

• We recommend performing all KO experiments using pooled populations, as clonal populations can have clonal effects independent of the specific KO. If the laboratory uses clonal KO populations, we recommend performing resistance assays on three independently generated KO clones to ensure that the effects are due to the KO of interest and not clonal variation.
• If using a pooled KO population, incomplete KO can result in resistant populations growing out in the ‘KO’ wells that express the secondary target of interest. We recommend only performing resistance assays on populations showing > 80% deletion of the secondary target.
• To determine whether incomplete KO of a secondary target is artificially skewing the analysis to underrepresent the overall effect of the KO, individual resistant wells can be expanded in the presence of drug and assessed for deletion of the secondary target by Western blotting. For example, we found that in H1975 cells that showed 85–90% *SOS2* deletion in pooled cultures, 4 / 37 osimertinib-resistant *SOS2*^*KO*^ wells showed SOS2 expression after expansion (see Theard et al. 2024^2^).

## Resource availability

### Lead contact

Further information and requests for resources and reagents should be directed to and will be fulfilled by the lead contact, Dr. Robert Kortum (robert.kortum@usuhs.edu).

### Technical contact

Requests for additional information about the protocol should be directed to Dr. Robert Kortum (robert.kortum@usuhs.edu).

### Materials availability

This study did not generate new, unique reagents.

### Data and code availability

There are no additional data associated with this protocol.

## Acknowledgments

This work was supported by funding from the 10.13039/100000002NIH (R01 CA255232 and R21 CA267515 to R.L.K. and P20 GM121316 to R.E.L.) and the 10.13039/100000090CDMRP Lung Cancer Research Program (LC180213 to R.L.K.). The funders had no role in the study design, data collection and interpretation, or the decision to submit the work for publication. The methods in this paper used data previously published by Sealover et al. in *iScience* (2024)^1^ and Theard et al. in *Molecular Oncology* (2024)^2^ to illustrate additional uses for the ISRA. The paper titled “SOS2 modulates the threshold of EGFR signaling to regulate osimertinib efficacy and resistance in lung adenocarcinoma”/Molecular Oncology 18(3) was published under a Creative Commons Attribution (CC BY) license. A link to this manuscript can be found at https://febs.onlinelibrary.wiley.com/doi/full/10.1002/1878-0261.13564. Data in Figure 4D are reproduced from this manuscript under the CC BY license.

The opinions and assertions expressed herein are those of the authors and are not to be construed as reflecting the views of Uniformed Services University of the Health Sciences or the United States Department of Defense.

## Author contributions

Conceptualization, N.E.S., J.M.H., P.L.T., and R.L.K.; methodology, N.E.S., J.M.H., P.L.T., D.C., A.J.L., and B.R.D.; formal analysis, N.E.S., J.M.H., D.C., P.L.T., and R.L.K.; writing – original draft, N.E.S., J.M.H., D.C., and R.L.K.; writing – review and editing, N.E.S.; funding acquisition and supervision, R.E.L. and R.L.K.

## Declaration of interests

The authors declare no competing interests.

## Supplemental information

Table S1. Excel template for ISRA scoring, related to step 5 Tab 1 has a blank template that can be used for scoring ISRA experiments. Tab 2 shows ISRA data for H1975 cells treated with increasing doses of osimertinib. Tab 3 shows data from tab 2 converted for entry into GraphPad Prism for plotting Kaplan-Meyer survival curves.
